# The Effect of Touch-Cure Polymerization on the Conversion and Hardness of Core Build-Up Resin Composites: A Laboratory Study

**DOI:** 10.3390/ma14206025

**Published:** 2021-10-13

**Authors:** Maria Dimitriadi, Aikaterini Petropoulou, Konstantinos Masouras, Maria Zafiropoulou, Spiros Zinelis, George Eliades

**Affiliations:** 1 Department of Biomaterials, School of Dentistry, National and Kapodistrian University of Athens, 115 27 Athens, Greece; mar.dimitriadi82@gmail.com (M.D.); maria.zaf28@hotmail.com (M.Z.); szinelis@dent.uoa.gr (S.Z.); 2 Department of Prosthodontics, School of Dentistry, National and Kapodistrian University of Athens, 115 27 Athens, Greece; aikatpetropoulou@gmail.com; 3 Department of Operative Dentistry, School of Dentistry, National and Kapodistrian University of Athens, 115 27 Athens, Greece; kmasoura@dent.uoa.gr

**Keywords:** core build-up composites, touch-cure polymerization, conversion, Martens hardness, ATR-FTIR

## Abstract

To improve the self-curing capacity and interfacial strength with dentine of dual-cured composite materials, touch-cure activators have been introduced. The aim of the study was to evaluate the effect of these activators on the hardness and conversion of dual-cured resin composite core build-up restoratives. The materials tested were Clearfil DC Core Plus (CF) and Gradia Core (GC) with the corresponding adhesives Clearfil S^3^ Bond Plus (for CF) and G-Premio Bond/G-Premio DCA activator (for GC). Disk-shaped specimens (n = 6/group) were prepared for the following groups: dual-cured, self-cured and self-cured in contact with the adhesive activators at the bottom surface. After a 3-week storage period (dark/dry/37 °C) the Martens hardness (HM) and degree of conversion (DC%) were determined for the previously mentioned groups and the top surfaces of groups in contact with the adhesives. A statistical analysis was performed by a one-way ANOVA and Holm–Sidak test per material and a Pearson’s correlation analysis (HM vs. DC%) at an α = 0.05. The self-cured specimens resulted in significantly lower HM and DC% values from the dual-cured group, as expected. However, in the presence of the adhesives with touch-cure activators, the conversion of the self-cured groups showed insignificant differences in HM and DC% from the dual-cured in both composite materials. The improvements on the bottom composite surfaces in contact with the adhesives did not extend to the entire specimen length. Nevertheless, improved interfacial curing may improve interfacial durability.

## 1. Introduction

Dual-cured resin composite core build-up materials are routinely used for the reconstruction of damaged tooth crowns with poor retention and resistance characteristics prior to a crown placement. Most of these materials are currently available in a flowable consistency containing light-curing catalysts and self-curing redox systems (i.e., amine-peroxide) for the adequate bulk curing of regions inaccessible to the activating light. However, a significant hysteresis of self-curing over dual-curing polymerization has been registered in many commercially available core build-up products affecting a series of properties with clinical implications such as the residual monomer, degree of conversion of C=C bonds, cross-linking density, hardness, flexural strength, elastic modulus and brittleness [1,2,3,4,5,6,7,8]. 

To mediate the adhesion of dual-cured core build-up composite materials with dental hard tissues, dual-cured adhesives have been introduced containing aryl sulphinate or aryl borate salts, which exert a synergic activation effect in contact with the composite and are known as a touch-cure activators [9]. This activation mode improves the degree of the C=C conversion of the etch and rinse adhesives in comparison with their light-cured analogues [10]. Moreover, it restores the amine protonization of self-curing composite redox systems induced by strong and mild simplified acidic adhesives [11,12,13]. However, concerns have been expressed about the low setting capacity of dual-cured adhesives without the synergic effect of chemical-curing resin luting agents [14] and incompatibility problems have been identified in several materials when using self-etch adhesives despite the self-curing activation [15]. 

Currently, dedicated simplified self-etch adhesives have been developed incorporating aryl sulphinate salts into the adhesive vial, thus reducing the application steps. Despite these developments, there is no direct information on the effect of the adhesive activators on the self-curing conversion of core build-up composites. 

The aim of the present study was to access the effect of a dual-cured and a dedicated simplified single-step self-etch adhesive on the hardness and self-curing conversion of dual-cured core build-up materials. The null hypothesis was that there is no effect of the adhesives on the properties of the corresponding core build-up materials and the alternative hypothesis was that a positive effect exists. 

## 2. Materials and Methods

The dual-cured resin composite core build-up materials and their corresponding adhesives with self-cure activation systems selected for the study are presented in Table 1. CB is a single-step self-etch adhesive incorporating a dual-cured activator to be used with a CF composite. GB is a self-etch universal adhesive to be mixed with the dual-cure activator, GD, and is used with a GC composite. These activators are representative of the currently available products for this category of restorative materials.

### 2.1. Specimen Preparation

**Figure 1:**
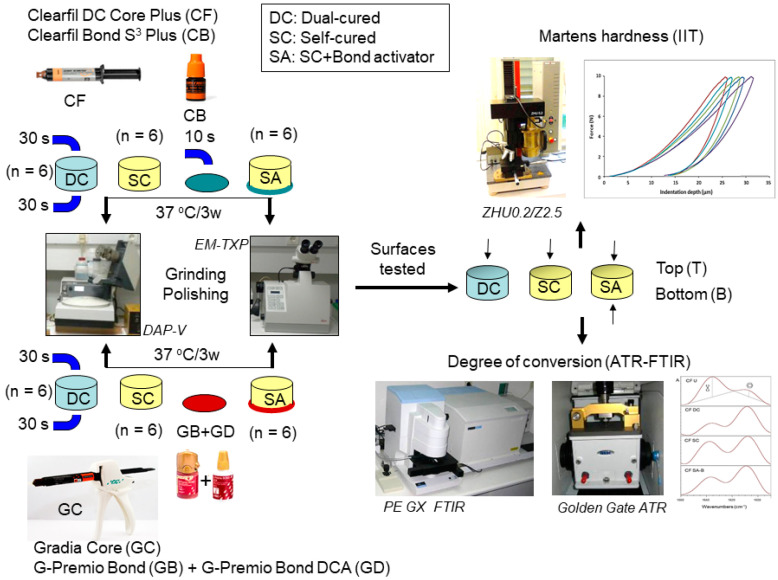
Schematic representation of the specimen preparation and the testing procedures of the study.

The specimen preparation and testing procedures used in the study are presented in Figure 1. 

Three groups of resin composite core build-up specimens were prepared (n = 6 × 3/product). The first group comprised dual-cured specimens (DC); the second, self-cured (SC) and the third, self-cured polymerized in contact with the dual-cure activators (SA). For the DC and SC groups, the mixed pastes were inserted into split Teflon ring molds (Ø = 10 mm, h = 4 mm) placed on microscopic glass slides, which were covered with transparent cellulose matrix strips and pressed with another set of strips and slides to remove the material excess. The specimens of the DC group were light-cured immediately after mixing for 60 s (2 × 30 s, top/bottom surfaces) with an LED curing unit (Radii Plus, SDI, Bayswater, Brisbane, Australia, 1500 mW/cm^2^ light intensity, standard exposure mode) whereas the specimens of the SC group were not irradiated. The specimens of the SA groups were prepared as follows: for the CF composite, a thin film of CB adhesive was placed on the bottom strip, air-dried for 5 s, light-cured for 10 s and then the composite was applied and self-cured. For the GC composite, a thin film of the mixture of the GB adhesive with the GD activator (1:1) was placed on the bottom strip, air-dried for 5 s and then the composite was applied and self-cured. All specimen groups were stored in dark and dry conditions at 37 °C for 3 weeks. They were then removed from the molds and the top surfaces of groups DC, SC and SA (SA-T) were wet-ground with 600–1000 grit-size SiC papers in a grinding/polishing machine (Dap-V, Struers, Ballerup, Denmark). The bottom surfaces of the SA groups in contact with the adhesive activators (SA-B) were subjected to controlled grinding to remove the yellowish adhesive layer (~10 μm maximum thickness) using a stereomicroscope-assisted milling/sawing/grinding system (EM TXP Target Surfacing System, Leica, Wetzlar, Germany) with 800 and 1000 grit-size SiC papers. All specimens were rinsed with water, air-dried with a strong stream of air (20 s) and their flat surfaces were used for testing. The DC and SC specimens were used as the controls; the SA-B specimens were used to determine the effect of the adhesive activators on the self-curing capability of the composites and the SA-T specimens to assess whether the adhesive activation extended throughout the entire specimen length.

### 2.2. Hardness Measurements

Martens Hardness (HM) measurements (n = 6/group) were performed with a universal hardness testing machine, a ZHU0.2/Z2.5 (Zwick Roell, Ulm, Germany), according to the ISO 14577-1 specification [16] for instrumented indentation testing (IIT). Force-indentation depth curves were recorded employing a Vickers indenter under a 9.8 N load, 0.5 mm/s (loading) and 0.1 mm/s (contact point and unloading) speed, 2 s dwell time and second-order polynomial zero-point determination. Three readings were taken from the surface of each specimen and the mean value served as the representative. 

### 2.3. Degree of Conversion Measurements

The specimens used for the hardness measurements were also employed for the assessment of the degree of conversion. The margins of the corresponding flat surfaces were pressed against the diamond reflective crystal (type III, 2 × 2 mm) of an attenuated total reflection (ATR) accessory (Golden Gate, Specac, Orpington, Kent, UK) attached to an FTIR spectrometer (Spectrum GX, PerkinElmer, Buckinghamshire, UK). The spectra were acquired under the following conditions: a 4000–650 cm^−1^ wavenumber range, 4 cm^−1^ resolution, 20 scans co-addition and ~2 μm depth of analysis at 1000 cm^−1^. The spectra of the uncured paste specimens recorded immediately after mixing (within 30 s at 23 °C vs. the nominal 5–6 min self-curing setting time of the products at 37 °C) were used as the unset controls. To verify the absence of adhesive film interferences from the composite surfaces analyzed, the spectra of the adhesives were recorded as before and compared via subtraction with the spectra of the corresponding composite materials to identify the peaks exclusively assigned to the adhesives. These peaks were then traced in the spectra of the SA-B groups as evidence of adhesive residues. For the degree of conversion measurements, the aliphatic C=C stretching vibrations at 1635 cm^−1^ were chosen as the analytical bands (AN) whereas the aromatic C..C stretching vibrations at 1605 cm^−1^ for the CF and the N–-H vibrations at 1545 cm^−1^ for the GC were selected as the reference bands (RF). The quantification was performed according to the equation:

Degree of Conversion% = 100 × [1 − (ApAN × AmRF/AmAN × ApRF)]

where A is the net peak absorbance height of the set (p) and unset (m) materials at the analytical (AN) and reference (RF) bands, respectively.

### 2.4. Statistical Analysis

The normality and equal variance of the values registered were assessed by Shapiro–Wilk and Brown–Forsythe tests. A statistical analysis of the Martens hardness and the degree of conversion between the groups tested (DC, SC, SA-T, SA-B) per material was performed by a one-way ANOVA and a Holm–Sidak post-hoc test. A Pearson’s product moment correlation analysis was used to evaluate the relationship between the two properties tested. For the statistical analysis, Sigma Plot v.14 software (Systat Software Inc., San Jose, CA, USA) was used at a 95% confidence level (α = 0.05).

## 3. Results

**Figure 2:**
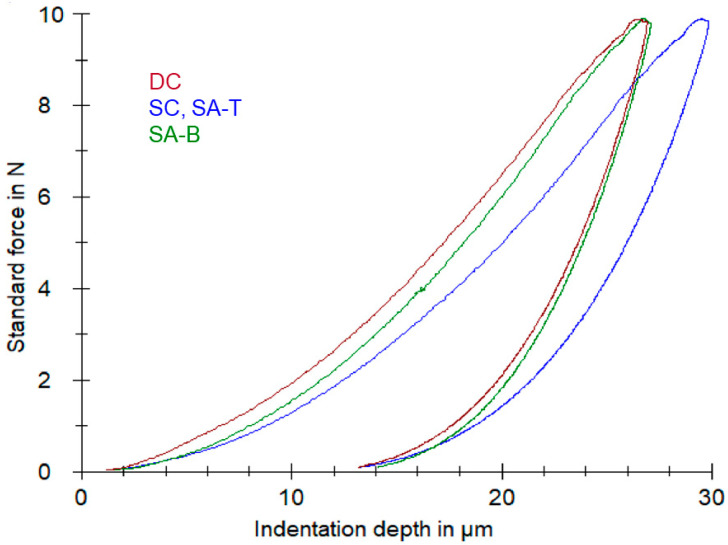
Force-indentation depth IIT curves for Clearfil DC Core Plus (CF) curing modes. DC: dual-curing; SC: self-curing; SA-T: self-curing, top surface with the adhesive activator (same with SC) and SA-B: self-curing, bottom surface in contact with the adhesive activator. The left peak shifting in DC and SA-B denotes an increase in the hardness.

The representative force-indentation depth graphs of the core build-up material for the curing modes tested are illustrated in Figure 2. The left curve peak shifting in DC and SA-B in comparison with SC and SA-T indicated an increased hardness in the former. Table 2 summarizes the results of the Martens hardness measurements. The data distributions passed normality (*p*-value: 0.062 for CF, 0.419 for GC; *p* > 0.05) and equal variance tests (*p*-value: 0.698 for CF, 0.186 for GC; *p* > 0.05). The one-way ANOVA demonstrated significant differences among the curing mode groups (*p* < 0.001; both materials). The ranking of the statistically significant differences was DC, SA-B > SC, SA-T for both materials (*p* < 0.05).

**Figure 3:**
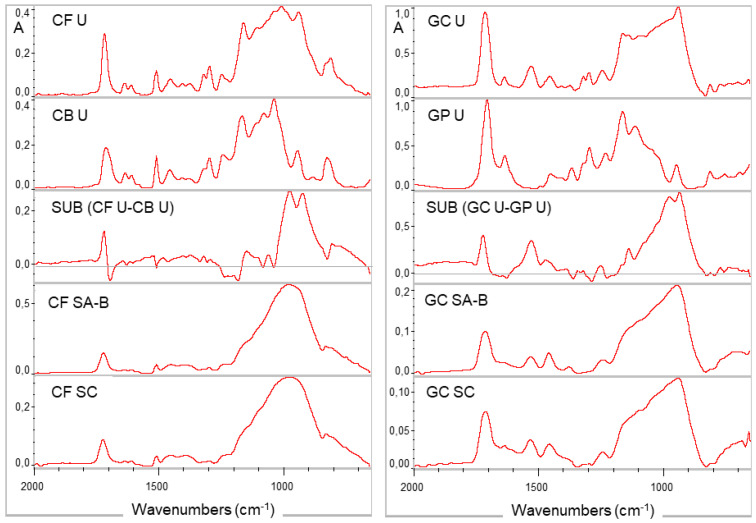
Expanded ATR-FTIR spectra of the composites (CF: Clearfil DC Core Plus; GC: Gradia Core) at an unset state (U), after self-curing without the adhesive activators (SC) and after self-curing with the adhesive activators (CB: Clearfil S^3^ Bond Plus; GP: G-Premio Bond mixed with a DCA activator) in contact at the bottom surfaces (SA-B). The spectra of the SA-B groups are free of adhesive spectroscopic interferences, which are resolved in the negative values of the subtraction spectra (SUB) of the unset adhesives (U) from the composites (absorbance scale, 2000–650 cm^−1^ wavenumber range).

**Figure 4:**
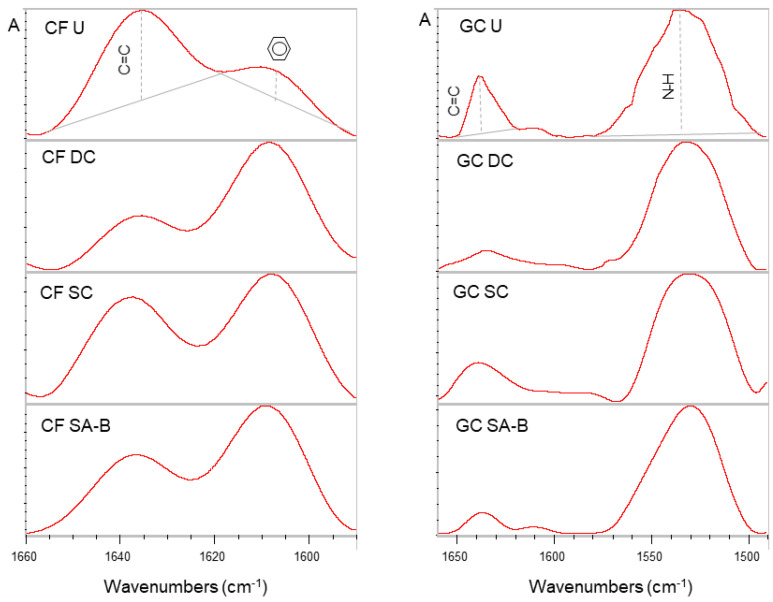
Expanded ATR-FTIR spectra of the unset (U), dual-cured (DC), self-cured (SC) and self-cured specimens in contact with the adhesive activators (SA-B) for Clearfil DC Core Plus (CF) and Gradia Core (GC) composites along with the analytical (C=C) and reference band (aromatic C..C or N–H) peak height annotations used for the degree of conversion measurements (absorbance scale, 1660–1590 cm^−1^ wavenumber range for CF and 1660–1490 cm^−1^ wavenumber range for GC).

The ATR-FTIR spectra of the SA-B surfaces were similar to the activator-free SC surfaces for each core build-up composite, confirming the efficient removal of the adhesive activators by the controlled target surfacing method (Figure 3). The representative spectra of the unset, DC, SC and SA-B groups used for the degree of conversion measurements are presented in Figure 4. The results of the percentage degree of conversion are presented in Table 3. The data passed normality (*p*-value: 0.689 for CF, 0.757 for GC; *p* > 0.05) and equal variance tests (*p*-value: 0.173 for CF, 0.643 for GC; *p* > 0.05). The one-way ANOVA demonstrated significant differences among the curing mode groups (*p* < 0.001 for CF and *p* = 0.003 for GC) with a ranking of significant differences of DC, SA-B > SC, SA-T for both materials (*p* < 0.05).

## 4. Discussion

The dedicated single-bottle self-etch adhesive CB and the dual-cured version of the self-etch adhesive GB improved the self-curing polymerization of the corresponding core build-up composite materials (CF, GC) in contact with the adhesive, rendering the previously recorded differences from the dual-curing mode insignificant. Therefore, the null hypothesis should be rejected and the alternative should be accepted. 

The results of the hardness and degree of conversion measurements confirmed the superior performance of the DC over the SC polymerization modes in both dual-cure core build-up restoratives, which agrees with the conclusions of a recent work [8] and previous studies [4,5,6]. These differences were registered after a prolonged specimen storage (3 weeks, dark/dry conditions at 37 °C), a period that included the contribution of delayed post-curing effects [17], which are especially important for slow-setting self-curing materials. As the effect of light-curing in several core build-up materials is limited to an upper 3–4 mm depth, self-curing is the mechanism that dominates material setting at greater depths [5,18], mostly associated with stress-bearing material hard tissue interfaces. 

Under the current experimental conditions, the use of the dual-cure adhesive activators restored the conversion and hardness values at the bottom surfaces of the self-cured core build-up materials in contact with the activators at the level of the dual-cured controls due to the interfacial initiation of polymerization. This curing mode, frequently referred to as a touch-cure mechanism [9], has been introduced to assist the bonding of dual-cured composites to dentine in regions with a reduced or no exposure to the activating light. The effect was further pronounced when the touch-cure activators of the adhesives contained photo-initiators and were subjected to extended irradiation [19]. Dual-cured adhesives have been also found to improve the bond strength of direct resin composites to dentine in comparison with their light-cured analogues, a finding possibly assigned to improved interfacial curing [20].

Although the positive effect of touch-cure activators has been documented for resin bonding to dentine and in several cases for the conversion of the adhesives *per se*, their influence on the conversion and properties of resin composites in contact with the adhesive has not been fully investigated so far. An ATR-FTIR spectroscopic analysis of dual-cured adhesive mixtures with resinous luting agents has elucidated the critical role of direct light-curing [21]. The same methodology has been recently used to assess the effect of a new Vanadium-based touch-cure primer on the setting of a resin luting agent [22]. However, such studies cannot differentiate the contributing phases (adhesive, luting agent) from the values recorded and apply for an undefined phase mix because the unset materials applied onto the ATR crystal demonstrate huge differences in viscosity and surface tension. Usually, a low viscosity non-irradiated adhesive film is placed first, air-dried and then covered by a medium viscosity flowable resin composite, which is pressed to level its free surface to a certain thickness. Under these conditions, the reproducibility of the superficial zone analyzed by ATR (<3 μm) may greatly vary per specimen depending on the extent of the phases mixed (i.e., complete displacement of the adhesive, creation of isolated adhesive or composite domains, diffusion of the adhesive into the composite). The method is even more challenging for application on irradiated adhesive films, the thickness of which usually exceeds the depth of the ATR analysis assuming a proper film setting without oxygen inhibition.

In the present study, the set adhesive films, as optically discriminated by their yellowish color, were removed from the bottom of the specimens by controlled grinding to expose the underlying composite surfaces. Although in pilot experiments the set adhesive films did not interfere with the Martens hardness measurements due to their very low hardness and thickness, they were removed from the bottom composite surfaces to allow for proper application of the surface-specific ATR analysis. To increase the sensitivity of the ATR method in probing the adhesive residues, the subtraction spectra between the unset materials (composite–adhesive) were recorded and the peaks exclusively assigned to the adhesives were traced on the ground composite surfaces, confirming the presence of adhesive-free surfaces. It has been postulated that surface grinding may increase the degree of conversion in resin composites by the thermal activation of residual C=C bonds [23]. The influence of this effect in the current experiment was negligible because the measurements were obtained after 3 weeks of storage at 37 °C, a period exceeding the shelf-life of free radicals available for any further polymerization [24]. The improved setting of the self-cured specimens in contact with the adhesive (SA-B group) documented by the ATR technique (2 μm analytical depth) extended into 30 μm (IIT indentation depth) as confirmed by the Martens hardness measurements. However, it did not extend to the entire specimen length as the top surface of the same specimens (SA-T group) demonstrated values similar to the self-cure group (SC). The results showed that the effects of the two touch-cure mechanisms tested on the composite base conversion were similar, regardless of the light-activation status (light-curing of the activator-containing CB adhesive, self-curing of the GB adhesive with the GA activator). Moreover, the mechanism was efficient for both composite materials with a different monomer composition (the Bis-GMA-based CF and the aliphatic UDMA-based GC) and reactivity [4,8]. Therefore, the previously documented higher bond strength to root canal dentine of core build-up materials combined with light-cured activator containing adhesives in comparison with their dual-cured versions, may be rather associated with the adhesive film properties than the conversion of the main resin composite restorative, [18]. The in-depth extension of the touch-cure mechanism in restorative resin composites is unknown. Recently, the effect of proprietary single-bottle touch-cure activators on the polymerization of resin composite luting agents (conventional or self-adhesive) has been evaluated employing the degree of conversion and nanohardness profiles as a function of the distance from the dentine composite interface. A positive effect was documented only for one primer extending 15 microns into a self-adhesive resin composite luting agent [25]. The dual-cured resin composite core build-up materials are more viscous and stronger than the composite luting agents with no acid-functionalized monomers. In such materials, the touch-cure activation mechanisms may further stabilize the tissue material interfaces and improve the biocompatibility of the adhesive restorative complexes.

For the hardness measurements, an IIT method was chosen to avoid optical and viscoelastic interferences such as the elastic recovery effect [26]. The profiles (top/bottom or cross-sectional) of the conventional hardness methods (Knoop, Vickers) initially introduced for the depth of the cure assessment in light-cured materials have been successfully employed for the testing of the self-curing capacity of dual-cured core build-up resin composites [2,5,27]. The moderate to strong correlations between the Martens hardness and the degree of conversion documented in the present experiment suggest that the differences in the Martens hardness per material can also be used as a curing indicator.

In the present study, the acidic self-etch adhesives were in contact with an inert substrate and not with dentine, which prohibits acidic monomer neutralization and may negatively affect the degree of conversion [28]. Therefore, measurements on dentine may further improve the interfacial conversion and the associated properties.

Undoubtedly, the depth of the adhesive-induced polymerization in dual-cured resin composites merits further investigation especially when new touch-cure activators are currently introduced with a complex chemistry [22]. The application of methods with a high spatial resolution for the assessment of the conversion and mechanical properties (i.e., Raman or FTIR microspectroscopy, microhardness and nanohardness) may provide important information on the efficacy of the emerging touch-cure activators in future studies.

## 5. Conclusions

Within the limitations of the current study, the following conclusion can be drawn. The self-curing capacity of the core build-up materials tested was significantly improved by the touch-cure mechanisms of the adhesives with dual-cure activators. The improvement resulted in values with insignificant differences from the dual-cured controls. However, the effect did not extend to the entire material length. 

## Figures and Tables

**Table 1:** The materials tested.

Product (Code)	Composition *	Manufacturer
Clearfil DC Core Plus (CF) Clearfil S^3^ Bond Plus (CB)	Bis-GMA, TEGDMA, hydrophilic aliphatic dimethacrylate, hydrophobic aromatic dimethacrylate, silanated BA-glass filler, silanated SiO_2_, Al_2_O_3_ filler, initiators, pigments, accelerators. (Filler: 74% w/52% v, 0.01–20 μm) Shade: dentine 10-MDP, Bis-GMA, 2-HEMA, hydrophilic aliphatic dimethacrylate, hydrophobic aliphatic methacrylate, colloidal SiO_2_, NaF, accelerators, initiators, ethanol, water (pH = 2.3)	Kuraray Noritake Dental Inc., Okayama, Japan
Gradia Core (GC) G-Premio Bond (GB) G-Premio Bond DCA (GD)	UDMA, NPGDMA, GDMA, TEGDMA, silanated Al-F-silicate glass, amorphous SiO_2_, TiO_2_, Fe_2_O_3_, MgO, initiators, accelerators. (Filler: 75% w) Shade: universal 10-MDP, 4-MET, MTDP, methacrylic acid ester, silica, acetone, water, photo-initiators (pH = 1.5). Ethanol, catalyst.	GC Corporation, Tokyo, Japan

* According to the manufacturers’ information. Bis-GMA: bisphenol glycidyl dimethacrylate; TEGDMA: triethylene-glycol dimethacrylate; 10-MDP: methacryloyloxydecyl dihydrogen phosphate; 2-HEMA: 2-hydroxyethyl methacrylate; UDMA: urethane dimethacrylate; NPGDMA: neopentyl glycol dimethacrylate; GDMA: glycol dimethacrylate; 4-MET: 4-methacryloxyethyl trimellitic acid; MDTP: methacryloyloxydecyl dihydrogen thiophosphate.

**Table 2:** Results of the Martens Hardness (means and SDs) Per Material and Curing Mode *.

Product	Martens Hardness (N/mm^2^)			
	DC	SC	SA-T	SA-B
CF	528 (14) ^a^	440 (22) ^b^	447 (22) ^b^	499 (12) ^c^
GC	568 (11) ^a^	493 (24) ^b^	495 (32) ^b^	530 (14) ^a^

* DC: dual-curing; SC: self-curing; SA-T: top surface of self-curing in contact with the adhesive activator; SA-B: bottom surface of self-curing in contact with the adhesive activator. The superscript letters show the means with no statistically significant differences between the curing modes per material (*p* > 0.05).

**Table 3:** Results of the Degree of C=C Conversion (%) Per Material and Curing Mode *.

Product	Degree of C=C Conversion (%)			
	DC	SC	SA-T	SA-B
CF	61.9 (3.6) ^a^	54.9 (2.2) ^b^	55.1 (2.5) ^b^	59.5 (2) ^a,b^
GC	59.2 (1.5) ^a^	54.7 (1.8) ^b^	54 (2.4) ^b^	57.6 (1.4) ^a,b^

* DC: dual-curing; SC: self-curing; SA-T: top surface of self-curing in contact with the adhesive activator; SA-B: bottom surface of self-curing in contact with the adhesive activator. The superscript letters show the means with no statistically significant differences between the curing modes per material (*p* > 0.05).

## Data Availability

Not applicable.
